# Prospects for Modeling Abnormal Neuronal Function in Schizophrenia Using Human Induced Pluripotent Stem Cells

**DOI:** 10.3389/fncel.2017.00360

**Published:** 2017-11-23

**Authors:** Iya Prytkova, Kristen J. Brennand

**Affiliations:** ^1^Department of Neuroscience, Icahn School of Medicine at Mount Sinai, line>New York, NY, United States; ^2^Friedman Brain Institute, Icahn School of Medicine at Mount Sinai, New York, NY, United States; ^3^Department of Genetics and Genomics, Icahn School of Medicine at Mount Sinai, New York, NY, United States; ^4^Icahn Institute of Genomics and Multiscale Biology, Icahn School of Medicine at Mount Sinai, New York, NY, United States; ^5^Department of Psychiatry, Icahn School of Medicine at Mount Sinai, New York, NY, United States

**Keywords:** human induced pluripotent stem cells, induced neurons, co-culture, organoids, schizophrenia

## Abstract

Excitatory dopaminergic neurons, inhibitory GABAergic neurons, microglia, and oligodendrocytes have all been implicated in schizophrenia (SZ) network pathology. Still, SZ has been a difficult disorder to study, not only because of the limitations of animal models in capturing the complexity of the human mind, but also because it is greatly polygenic, with high rates of variability across the population. The advent of patient-derived pluripotent stem cells and induced neural and glial cultures has brought hope for modeling the molecular dysfunction underlying SZ pathology in a patient-specific manner. Here I review the successes of the patient-specific induced cultures in generating different cell types for the study of SZ, with special emphasis on the utility of co-culture techniques, both two- and three-dimensional, for modeling network dysfunction in disease.

## Introduction

Schizophrenia (SZ) is a common debilitating psychiatric disorder which affects approximately 1% of the world population and accounts for a significant socio-economic burden (Rössler et al., 2005). The disease is characterized by the presence of positive symptoms, such as hallucinations, delusions, and paranoia, and negative symptoms, such as social withdrawal and flattened affect; SZ is also marked by cognitive deficits such as disorganized thoughts and difficulty concentrating (Barnhill, 2013). The symptoms may appear in a variety of constellations. Additionally, individuals with SZ have a high risk of suicide, high prevalence of substance abuse, and a high rate of homelessness (Rössler et al., 2005). Despite being recognized as early as 1896 (Rössler et al., 2005), disease etiology has remained elusive. The rate of heritability has been estimated to be around 80%, but the concordance rate among monozygotic twins is around 50% (Cardno et al., 1999), indicating that genetic contribution alone is insufficient to fully account for disease risk. Moreover, genome wide associated studies (GWAS) by the Schizophrenia Working Group of the Psychiatric Genomics Consortium (2014) have identified over one hundred loci associated with the disease, consistent with the clinical heterogeneity of the disorder.

## Network Dysfunction In Schizophrenia

Overwhelming genetic evidence now demonstrates that SZ is a complex genetic disorder (Schizophrenia Working Group of the Psychiatric Genomics Consortium, 2014) reflecting cumulative risk from more than 100 rare and common variants. Although the genetic and symptomatic heterogeneity of SZ implicates a wide variety of neurotransmitter, neuroimmune, neuroanatomical, and neurodevelopmental processes (reviewed xx), the precise cell type(s) responsible for the cell autonomous defects underlying the earliest disease processes remain unresolved. Historically, the effectiveness of D_2_-receptor antagonists as anti-psychotics shaped much of the thinking surrounding the neurobiological mechanisms of SZ (Delay et al., 1952; Carlsson et al., 1957); while successful in some cases at alleviating the positive and, to a lesser extent (Kirkpatrick et al., 2001), negative symptoms of SZ, these drugs produce a plethora of side effects which lead to high rates of discontinuation (Lieberman et al., 2005). Glutamatergic signaling via *n*-methyl-D-aspartate receptor (NMDA-R) has also been repeatedly linked to disordered cognition and sensory processing (for review see Kantrowitz and Javitt, 2012); NMDA-R antagonists, such as phencyclidine and ketamine, mimic some cognitive and behavioral symptoms of SZ (Kehrer et al., 2008). Another possibility is that the loss of NMDA receptors on GABAergic interneurons leads to hyper-activation of cortical forebrain neurons (Olney et al., 1999), thus leading to an overall excitation/inhibition imbalance (Homayoun and Moghaddam, 2007). Since glutamatergic signaling on GABAergic interneurons has an important regulatory function on the activity of dopaminergic neurons, it seems reasonable that genetic variants impacting any (or all) of these cell types could lead to convergent elements of symptomology across genetically heterogeneous patients.

There is a strong neurodevelopmental component to SZ risk (Weinberger, 1995). Most cases of SZ are diagnosed in adolescence or young adulthood (Lewis and Lieberman, 2000), although there are rare cases of childhood onset (Asarnow, 1994). During these stages of development, extensive synaptic pruning and overall loss of gray matter occurs (Huttenlocher and Dabholkar, 1997). Neuroimaging studies have shown a loss of frontal and hippocampal volume in SZ patients; post-mortem tissue analyses have further identified decreased spine density and synaptic connectivity (Glantz and Lewis, 2000; McGlashan and Hoffman, 2000; Faludi and Mirnics, 2011). Microglia and oligodendrocytes have also been implicated in SZ pathology, due to their roles in synaptic pruning (Paolicelli et al., 2011) and cell-to-cell communication (Tkachev et al., 2003), respectively.

The limitations of animal models in re-capitulating human development, the heterogeneity of this complex genetic disorder, and the variable response to pharmacological therapies underscore the necessity for modeling SZ in human cells. Neurons and glia derived from human induced pluripotent stem cells (hiPSCs) offer an opportunity to investigate the complex cellular and molecular interactions involved in SZ. Despite the fairly recent advent of hiPSCs in Takahashi et al. (2007), numerous advances have facilitated improved reprogramming of patient somatic cells into neurons, either directly or via a pluripotent stem cell intermediate. Below I review the available protocols for generating various neuronal cultures, with a focus on the application of each method to modeling cellular dysfunction in SZ. Finally, I address current limitations and future strategies to improve upon this approach.

## Patient hiPscs-Derived Brain Cells for Modeling Schizophrenia

Cellular and molecular dysfunction in SZ has traditionally been studied by examining post-mortem patient brain tissue or through animal models. Post-mortem tissue studies are limited by degradation of key biomolecules (i.e., RNA, DNA, proteins, epigenetic marks) due to delays between death and sample processing (Ferrer et al., 2008). On the other hand, animal models carry the benefit of *in vivo* manipulations at different developmental stages and isogenic controls. However, animal models of SZ rely either on targeted lesions, administration of psychotropic drugs such as phencyclidine, or on manipulating a single gene associated with the disorder (Brennand and Gage, 2011; Flores et al., 2016). These approaches have limited etiological relevance, given the highly polygenic nature of SZ discussed above. By contrast, patient hiPSC-derived neurons and glia offer a unique opportunity to investigate the full genetic landscape contributing to SZ while monitoring neural development (**Table 1**).

**Table 1:** Recent human induced pluripotent stem cell (hiPSC)-based studies of schizophrenia.

Mutation	Cell type	Reprogramming method	Phenotype	Reference
22q11.2	Glutamatergic neurons	Directed differentiation (WNT3A, BDNF, GDNF, cAMP, IGF1)	—	Pedrosa et al., 2011
4bp deletion in *DISC1*- frameshift	hiPSC	Integration-free episomes	—	Chiang et al., 2011
Not known	Forebrain (glutamatergic and GABAergic) neurons	Tetracycline-inducible lentivirus (*OCT4, SOX2, KLF4, c-MYC, LIN28*)	Decreased neuronal connectivity, increased *NRG1* expression; Loxapine rescue	Brennand et al., 2011
shRNA NRXN1 knock-down	Neurons	Pggy-Bac transposon (OCT4, SOX2, KLF4, c-MYC), then directed differentiation	Deficits in astrocyte generation, perturbations in cell adhesion and neuron differentiation pathways	Zeng et al., 2013
15q11.2	NPCs	Integration-free episomes or sendai virus	Defects with apical polarity and adherent junctions	Yoon et al., 2014
4bp deletion in *DISC1*- frameshift	Forebrain glutamatergic neurons	Integration-free episomes	Increased soma size and total dendritic length in immature neurons, decreased SV2++ puncti, frequency of sEPSCs and synaptic vesicle release	Wen et al., 2014
Not known	Hippocampal dentate gyrus granule neurons	Tetracycline-inducible lentivirus (*OCT4, SOX2, KLF4, c-MYC, LIN28*)	Deficits in generation of DG granule neurons, decreased neuronal activity and sEPSC frequency and amplitude	Yu et al., 2014
22q11.2	Neurons	Integration-free episomes	Recapitulation of mRNA and miRNA expression pattern expected of 22q11.2 haploinsufficiency	Zhao et al., 2015; Lin et al., 2016
NRXN1 hz deletion	Glutamatergic neurons	Tetracycline-inducible lentivirus (NGN2) in hESC	Decrease in mEPSC frequency and evoked neurotransmitter release; increase in synaptic scaffolding protein CASK	Pak et al., 2015
Not known	NPCs	Tetracycline-inducible lentivirus (*OCT4, SOX2, KLF4, c-MYC, LIN28*)	Aberrant migration, increased oxidative stress [mitochondrial membrane potential (MMP)]	Brennand et al., 2015

### Neural Cultures

Several groups have generated heterogeneous neuronal populations from a variety patient derived hiPSCs, focusing either on genetically defined (Chiang et al., 2011; Pedrosa et al., 2011; Wen et al., 2014) or unknown genetic (Brennand et al., 2011, 2015) patient cohorts. Chiang et al. (2011) were the first to report hiPSCs lines from three SZ patients harboring DISC1 mutations, which have been long implicated in SZ and other neuropsychiatric disorders, including autism and major depression. Although this mutation is known to occur in only two rare family pedigrees, the DISC1 protein is thought to bind many proteins involved in neuronal development and synapse formation (Duan et al., 2007; Porteous et al., 2014), potentially providing broader insights into disease mechanisms. Wen et al. (2014) advanced this work by generating fore-brain like neurons from SZ patients with a *DISC1* mutations as well as isogenic hiPSC lines, correcting mutations in one of the patients and introducing frameshift deletions in DISC1 into control hiPSCs. The corrected isogenic lines showed an improvement in synaptic functionality, as evidenced by the increase in synaptic markers (co-localization of SYN1 and PSD95) and increased amplitude and frequency of spontaneous synaptic currents, while the mutated isogenic lines showed a deficit in synaptic activity. The researchers then performed RNA-seq to gain understanding of the affected molecular pathways, and, for the first time, showed differential expression in DISC1-interacting proteins. Moreover, they identified differential regulation of several genes coding for presynaptic proteins (including neurexin 1, synaptophysin, and synaptoporin) and transporters (including MEF2C), implicating mutant DISC1 as a hub of transcriptional regulation (Wen et al., 2014).

Several copy number variations (CNVs) have been associated with increased SZ risk, including 22q11.2, 15q11.2, and NRXN1 loci (for review see St Clair, 2009). HiPSC studies of 22q11.2 deletions, found in approximately 1% of SZ cases (Bassett and Chow, 2008), have identified differential gene expression in pathways regulating cell cycle and development (Lin et al., 2016) as well as impaired neurite outgrowth and cellular migration (Toyoshima et al., 2016). Investigation of patient hiPSCs with a 15q11.2 microdeletion revealed deficits in apical polarity and adherens junctions due to CYFIP1 haploinsufficiency; genetic association analyses on these hiPSCs uncovered an epistatic interaction between a WAVE signaling mediator (involved in cytoskeleton development) and CYFIP1 (Yoon et al., 2014). Finally, NRXN1 expression was reduced in human embryonic stem cells (hESCs) by heterozygous deletion (Pak et al., 2015) and hiPSCs by shRNA (Zeng et al., 2013). Heterozygous mutant neurexins resulted in impaired neurotransmitter release and elevation of CASK (synaptic scaffolding protein) (Pak et al., 2015), and half reduction in NRXN1 expression led to changes in gene expression of cell adhesion and neuron differentiation pathways (Zeng et al., 2013). These studies highlight the utility of *in vitro* studies for manipulating human genes in human cells and generating human isogenic controls.

By contrast, Brennand et al. (2011) took a more generalized approach and generated hiPSC-derived neurons from four SZ patients who lacked a unifying genotype, but were selected on the basis of the likelihood of a genetic component to their disease (as supported by relatives affected by psychiatric disease or early age of onset). The group generated a mix of excitatory, inhibitory, and dopaminergic neurons, evidenced by the presence of VGLUT1, GAD67, and tyrosine hydroxylase, respectively. These patient-derived neurons formed fewer processes, exhibited decreased neuronal connectivity (assayed by *trans*-neuronal spread of rabies virus) and decreased spine density (Brennand et al., 2011). Most strikingly, many of the gene expression pathways perturbed in SZ hiPSC neurons were also detected in immature neural progenitor cells, indicating that these pathways were altered even before the establishment of post-mitotic neurons (Brennand et al., 2015). Overall, these findings mimic the decreased neuronal connectivity and synaptic function shown in post-mortem tissue studies (16, 33–35).

### Co-culture Systems

The above investigations demonstrate the utility of patient hiPSC-derived neurons for modeling cellular interactions, gaining insight in the impact of a given mutation on said interactions, and elucidating the molecular mechanisms responsible. Most importantly, this is achieved using human cells, thus providing much more translational value than animal models. Despite these advantages, a number of issues surround induced neural protocols (see Engel et al., 2016), especially in the context of modeling synaptogenesis and network dysfunction. First, gene expression studies indicate hiPSC neurons best resemble fetal brain tissue (Brennand et al., 2015). Second, hiPSC neurons require extensive cultivation periods of up to 7 months (Maroof et al., 2013; Nicholas et al., 2013; Suzuki and Vanderhaeghen, 2015). Moreover, pure neuronal cultures are limited in their ability to accurately re-capitulate network functionality, since glial cells play an important role in regulating neuronal activity and establishing myelination.

Several groups have developed astrocyte-neuron co-culture systems, using both rodent and human astrocytes with human iPSC-derived neurons, with promising results for maturation and network activity (Tang et al., 2013; Muratore et al., 2014; Odawara et al., 2014; Kuijlaars et al., 2016). Indeed, the notion that astrocytes enhance synapse maturation is well established from rodent cell cultures (Araque and Perea, 2004). Odawara et al. (2014) utilized multiple electrode array (MEA) plates to co-culture rat astrocytes with hiPSC-derived neurons and found increased electrophysiological activity after 3 weeks of plating, with neurons continuing to increase activity for more than 3 months after plating. Muratore et al. (2014) performed a direct comparison of several neuronal differentiation protocols and demonstrated a significant increase in vGLUT1 expression by day 40 in neurons cultured on mouse astrocytes, pointing to accelerated maturation of excitatory neurons. Kuijlaars et al. (2016) were the first to utilize astrocyte co-culture with solely human cells in 2016. Using commercially available fetal astrocytes, the group observed synchronized calcium oscillations in hiPSC-derived neurons 3–4 weeks after final plating, indicating sustained network activity (Kuijlaars et al., 2016).

Although astrocyte co-cultures have yet to be utilized to model neuropsychiatric disease, these studies are very promising, particularly the human-only system employed by Kuijlaars et al. (2016). Human neuron-astrocyte co-culture systems may be useful not only for improved neuronal maturation but also for elucidating astrocyte contribution to the disease state, especially taking into consideration the role of astrocytes in modulating synaptic connection and signaling (Goudriaan et al., 2014). Post-mortem tissue studies have shown decreases in astrocyte density in certain regions (Williams et al., 2013) and increases in astrocyte markers and neuroinflammation in a subset of patients (Catts et al., 2014). Co-culturing astrocytes and neurons derived from the same patient may more closely mimic the cellular and molecular dynamics of the individual disease state. Additionally, co-culturing patient astrocytes with healthy control neurons and healthy control astrocytes may help elucidate the contribution of each cell type to SZ etiology.

Oligodendrocyte dysfunction and myelin deficits have also been heavily implicated in SZ by genome association and post-mortem studies (Burns et al., 2003; Davis et al., 2003; Tkachev et al., 2003; Takahashi et al., 2011; Goudriaan et al., 2014; Schizophrenia Working Group of the Psychiatric Genomics Consortium, 2014). Specifically, volume reduction in myelin observed in SZ patients is consistent with deficits in neuronal communication and synaptic plasticity thought to underlie SZ pathology (reviewed by Takahashi et al., 2011). A hiPSC model of patient oligodendrocytes and healthy neurons would allow to investigate the precise consequences of patient-specific variation in oligodendrocyte genes on myelin formation, interactions with neurons, and synaptic plasticity. Additionally, given the well-established role of myelin in neuronal maturation (Hasegawa et al., 1992), co-culture with oligodendrocytes will likely improve the immature state of current neural hiPSC cultures. Although to date no co-culture system of hiPSC-derived oligodendrocytes and neurons has been reported, oligodendrocytes have been successfully generated from hiPSCs (Livesey et al., 2016; Ehrlich et al., 2017). Most excitingly, Ehrlich et al. (2017) demonstrated successful integration of hiPSC-derived oligodendrocytes into mouse brains, giving hope that this myelination can be reiterated *in vitro* with human cells.

Microglia mediate synaptic pruning during adolescence (Paolicelli et al., 2011) (although see Sekar et al., 2016 for neuronal contribution to synaptic pruning in SZ) and respond to immune threats by producing inflammatory cytokines (Kreutzberg, 1996); accordingly, abnormal pruning (Feinberg, 1982) and neuroinflammation (Ashdown et al., 2005; Monji et al., 2013) are thought to contribute to the etiology of SZ. Studying these functions in a hiPSC system would allow scientists to mix and match patient and control microglia and neurons, in order to determine the driving force behind abnormal cellular connectivity and function. Critically, microglia-like cells have been successfully generated from hiPSCs by several groups in the last year (Muffat et al., 2016; Abud et al., 2017; Pandya et al., 2017). These cells resemble fetal and adult human microglia in their gene expression patterns and show phagocytic activity *in vitro* and *in vivo*. A co-culture application for modeling synaptic pruning would benefit from more mature, myelinated neurons; however, hiPSC microglia show potential for modeling inflammatory activation early in development in a co-culture system.

### Modeling Regional Specificity and Circuitry

Although several protocols bias hiPSC differentiation into region-specific neurons, these methodologies fall far short of generating defined circuitry. Typically, hiPSCs are first transformed into neural progenitor cells (NPCs) through dual SMAD inhibition, which involves antagonism of bone morphogenic protein (BMP) and transforming growth factor beta (TGF-β) (Chambers et al., 2009). Such directed differentiation into NPCs (in contrast to allowing hiPSC colonies to differentiate in neural medium as in the studies by Brennand et al. (2011) and Pedrosa et al. (2011) significantly reduces variability in the subsequently derived neurons. The NPCs are then subjected to treatment with growth factors found in distinct regions of the developing brain. Thus, scientists have generated neurons specific to the medial and caudal ganglionic eminences (sites of GABAergic neuron development) (Ahn et al., 2016), cerebral cortex (Shi et al., 2012), and the dentate gyrus (Yu et al., 2014). Although directed-differentiation protocols are thought to more closely mimic *in vivo* development, the protracted timeline poses a major disadvantage. Viral-based induction strategies have been utilized to overexpress exogenous transcription factors to produce excitatory (Ho et al., 2016) and cortical inhibitory neurons (Colasante et al., 2015); however, these strategies carry the caveat of additional artificiality and may fail to affect all of the transcription factors necessary to induce regional specificity (see Chanda et al., 2013 and Pak et al., 2015 for evidence on same phenotype across different cell culture methods).

Even with regional specificity and micro-circuit modeling, two-dimensional cultures fail to recapitulate the three-dimensional organization of the brain and circuits therein. The ongoing development of three dimensional neural culture (alternatively called brain organoids or cortical spheroids) shows significant promise for modeling network activity and circuit organization. Paşca et al. (2015) derived cortical spheres containing non-reactive astrocytes and functional cortical neurons with laminated organization. Qian et al. (2016) designed a miniature bioreactor system to ensure proper circulation of nutrients in media and produced region-specific organoids that mimicked forebrain, midbrain, and hypothalamus expressing appropriate markers and resembling respective cytoarchitecture. Most recently, Birey et al. (2017) generated and fused together pallium- and subpallium-resembling organoids to model development and migration of inhibitory neurons. After 3 weeks of contact between the pallium and subpallium (ventral forebrain) organoids, inhibitory neurons from the subpallium sphere migrated into the pallium sphere, yielding functional excitatory and inhibitory post-synaptic connections. Creation of such chimeric organoids is an exciting prospect for modeling developmental cellular migration and connectivity in a 3D system.

Although organoids have yet to be applied to SZ, their potential for modeling neural developmental and electrophysiological anomalies is obvious. Much work remains, however, to adequately model circuit development and contributions of non-neural cells to SZ pathology. Currently, brain organoids contain only relatively immature neurons and non-reactive astrocytes, with oligodendrocytes and microglia still lacking. Potential injection of hiPSC-derived oligodendrocytes and microglia (discussed in the section “Co-culture System”) has promise for incorporation into organoid tissue, and the potential to recapitulate exciting *in vivo* findings that SZ patient hiPSC-derived glial cells function differently when injected into a mouse brain (Windrem et al., 2017), but differences in growing conditions and developmental requirements still pose major challenges *in vitro*.

While there is solid evidence that the neuropathologies of SZ often precede symptom onset, there are also clear adolescent neurobiological developmental processes (GABAergic maturation, excitatory synaptic pruning, microglia- and astrocyte-mediated neuroinflammation) and environmental exposures (cannabis abuse, stress) that can accelerate or exacerbate genetic risk (well-reviewed van Os et al., 2010). Moving forward, there is an urgent need to add further complexity to our hiPSC-based models in order to model these additional processes. Such efforts will involve not just improving the complexity of cell types, regional patterning and circuit formation, but also the restoration of critical features of “age” and “maturity” to hiPSC-derived cultures. While reprogramming of fibroblasts into hiPSCs erases age-related epigenetic marks and gene expression profiles, these can be at least partially maintained by inducing patient somatic cells directly into neurons (Mertens et al., 2015; Huh et al., 2016). Moreover, some environmental insults may be relatively easy to introduce, such as maternal infection/stress during development (Boksa, 2008), cannabis use (Henquet et al., 2005) or hypoxia during delivery (Cannon et al., 2002), while others such as trauma from childhood abuse and neglect (Read et al., 2005) may only be vaguely approximated by stress-related molecules such as corticosteroids or inflammatory cytokines.

## Concluding Remarks

Despite being in a nascent stage of methodological development, hiPSC-derived neurons show promise for modeling molecular and cellular deficits of SZ. Because of the complex etiology and great genetic variability of the disorder, a patient-specific approach may be illuminating from a mechanistic perspective as well as for drug screening. Already, hiPSC-derived neurons have recapitulated deficits in synaptic maturation and connectivity. Furthermore, advances in generating organoids show promise for modeling network activity in three dimensions, thus better recapitulating brain development and circuitry.

Of course, many limitations accompany the use of tissue culture to model psychiatric disease. Aside from being limited to molecular and cellular phenotypes with no recourse for assessing behavioral consequences, current methods produce immature neurons, limiting studies to the developmental predisposition of disease rather than the actual disease state. Nonetheless, modeling predisposition may uncover targets for preventative therapeutic interventions for patients with a high familial risk of psychiatric disease.

Given the developmental underpinnings and genetic heterogeneity of SZ, hiPSC cultures should complement animal models. Overall, subtype specific hiPSC-derived neuronal and glial (astrocyte, oligodendrocyte, and microglia) culture methods are rapidly evolving. Improved neuronal maturation, combined with novel three dimensional cultures, present an exciting opportunity to investigate circuit function and connectivity in patient-specific cells.

## Author Contributions

IP gathered references and wrote the manuscript. KB revised the manuscript and provided additional references.

## Conflict of Interest Statement

The authors declare that the research was conducted in the absence of any commercial or financial relationships that could be construed as a potential conflict of interest.
